# How Large Is the Role of Emotion in Judgments of Moral Dilemmas?

**DOI:** 10.1371/journal.pone.0154780

**Published:** 2016-07-06

**Authors:** Zachary Horne, Derek Powell

**Affiliations:** 1 Department of Psychology, University of Illinois at Urbana-Champaign, Urbana, Illinois, United States of America; 2 Department of Psychology, University of California Los Angeles, Los Angeles, California, United States of America; Illinois Institute of Technology, UNITED STATES

## Abstract

Moral dilemmas often pose dramatic and gut-wrenching emotional choices. It is now widely accepted that emotions are not simply experienced alongside people’s judgments about moral dilemmas, but that our affective processes play a central role in determining those judgments. However, much of the evidence purporting to demonstrate the connection between people’s emotional responses and their judgments about moral dilemmas has recently been called into question. In the present studies, we reexamined the role of emotion in people’s judgments about moral dilemmas using a validated self-report measure of emotion. We measured participants’ specific emotional responses to moral dilemmas and, although we found that moral dilemmas evoked strong emotional responses, we found that these responses were only weakly correlated with participants’ moral judgments. We argue that the purportedly strong connection between emotion and judgments of moral dilemmas may have been overestimated.

## Introduction

How do we decide whether an act is morally right or wrong? Though this question has a long history, the nature of the controversies surrounding moral decision-making has not fundamentally changed. Historically, there has been debate between philosophers who stressed the role of reason and deliberation in moral judgment (e.g., [1]) and those who argued that moral judgments are driven by emotional processes (e.g., [2–3]). These contrasting emphases are also evident in the course of psychological research on moral judgment. Early investigations were chiefly concerned with how morality was shaped through cognitive development (e.g., [4]). More recently however, a great deal of research has focused on the role of emotion in moral decision-making (e.g., [5–12]). These and other recent insights into the psychological processes involved in moral judgment have reinvigorated normative ethical debates about our moral obligations to ourselves and others (e.g., [9, 13–15]).

Theories of moral judgment have tended to emphasize the influence of either reason or emotion to moral judgment. However, it is quite likely that both of these capacities play a role in everyday moral evaluation. The roles of both of reason and emotion are integrated in the dual-process theory of moral judgment [7,16]. According to the dual-process theory, cold reasoning processes are recruited when making utilitarian moral judgments, but these judgments can be preempted by hot affective processes that lead people to make deontological moral judgments. Contemplating the violation of a moral rule elicits a strong negative emotional reaction that tends to elicit disapproval toward the violation. However, when violating the rule would bring about a better moral outcome, this prepotent response can be overridden by deliberative processes, leading to utilitarian approval for the action. The signatures of these two processes are thought to be evident in the so-called *personal-impersonal* distinction: researchers have found that people are less likely to approve of sacrificing one person to save others if a dilemma requires an “up-close-and-personal” action, such as physically pushing someone to their death, than if a dilemma requires an action that operates at greater distance, such as flipping a switch that leads to someone’s death. The dual-process theory has become very influential within the field of moral psychology over the last decade, and it is now widely accepted that people are less likely to approve of personal violations because they evoke strong emotional reactions compared to impersonal actions (e.g., [6, 7, 17–21], but see [22]).

### Reexamining the relationship between emotion and judgment in moral dilemmas

The largest and most widely-cited body of evidence for the role of emotion in judgments of moral dilemmas, and for the dual-process theory, has come from research examining people’s judgments about a single battery of moral dilemmas (henceforth, *the standard battery;* e.g., [6, 7, 17, 19, 20]). Most prominently, several neuroimaging studies have examined people’s judgments about dilemmas taken from the standard battery. For instance, in two studies, Greene et al. [6, 7] found increased activation in brain areas associated with emotion when participants made judgments about personal dilemmas, and increased activation in areas associated with reasoning processes when they considered impersonal dilemmas (but see [23]). In other studies, researchers demonstrated similar effects using psychophysiological measures of affect (e.g.,[20]) and when examining clinical populations with ventromedial prefrontal cortex lesions (an area of the brain thought to be critically involved in emotion and emotion regulation; e.g., [17, 19]).

However, there are problems with the standard battery [24–27]. Of particular concern is the fact that personal dilemmas in the standard battery more often involve physically harming a moral patient than do impersonal dilemmas. In fact, it appears that all of the personal dilemmas in the standard battery involve physical harm, whereas only half of the impersonal moral dilemmas do. This is potentially problematic, as the harmfulness of an action ought to be orthogonal to its up-close-and-personal nature. What’s more, it appears that personal dilemmas in the standard battery tend to involve more graphic and grisly descriptions of harm than do impersonal dilemmas, even when focusing only on impersonal dilemmas involving harm. For example, personal dilemmas ask participants to consider cutting off a man’s head, smothering a baby, or subjecting children to painful medical experiments. In contrast, impersonal dilemmas ask participants to consider venting deadly fumes into a room or voting for a new environmental policy that will harm people.

Researchers using the standard battery have often argued that the “closeness” of personal moral actions elicits a strong negative emotional reaction that in turn leads participants to make deontological moral judgments. Yet, one possibility is that researchers have observed stronger emotional reactions to personal dilemmas because the personal dilemmas in the standard battery more often involved grisly and harmful actions than did the impersonal dilemmas, and not because of the closeness of personal actions. If so, then prior studies may have only shown that graphic descriptions of harmful acts are emotionally salient. Thus, many studies taken to provide evidence for the dual-process theory may not provide a strong test of the central claim of the theory. That is, it is unclear whether emotional responses explain the difference in people’s judgments about personal and impersonal dilemmas or whether the observed differences are due to confounds in the stimuli.

Setting aside concerns about the standard battery, there is little work on precisely which emotions are involved in judgments of moral dilemmas. Further, there is no work to our knowledge demonstrating the causal strength of these emotional reactions. There is compelling evidence that disgust and anger are elicited in judgments about norm violations such as committing incest or suicide [5, 8–12, 28], but the neuroimaging studies that provide evidence for the role of emotional processes in moral judgments have not directly measured which emotions are involved in judgments of moral dilemmas.

In addition, very little work has examined the extent to which emotional processes are causally related to the moral judgments of neurotypical individuals (but see [19]). Whereas a number of researchers have argued that incidental emotional states can affect judgments of simple norm violations (e.g., [5, 12] but see [28]), attempts to demonstrate these effects on judgments about moral dilemmas have produced inconsistent results (e.g., [29–32]). Meanwhile, reaction time data (e.g., [6, 7; 33]) and experiments examining speed-pressure [34, 35] and cognitive load manipulations [16, 35] suggest that deliberative reasoning is crucial for utilitarian judgments—utilitarian judgments are sometimes slower, and seem to be impaired by speed-pressure and increased cognitive load—however, these findings cannot determine whether characteristically *emotional* processes produce deontological judgments. Rather, the current data only suggest that deontological judgments are produced by some sort of automatic or intuitive process. If the automatic processes involved in moral judgment are truly affective processes, then their operation ought to be accompanied by the qualitative experience of emotion, which is most easily measured by asking participants to report their emotional experiences.

Altogether, the current state of the literature suggests that more research is necessary to establish the role of emotion in judgments of moral dilemmas. If confounds in the standard battery affect participants’ emotional reactions, then it needs to be determined whether prior findings—that is, those demonstrating a connection between emotions and judgments of moral dilemmas—were a result of these confounds or of genuine *affective* differences between personal and impersonal dilemmas. In addition, existing research does not tell us what types of emotional responses are involved in judgments of moral dilemmas, nor does it inform us as to the strength of the relationship between people’s emotional responses and their moral judgments. Thus, we aim to reassess the extent to which emotional responses explain the difference between personal and impersonal judgments when the confounds in the standard battery are eliminated, and importantly, what specific emotions explain this difference.

We should point out that, as Greene et al. [36] have noted, the value of the dual-process theory of moral judgment does not depend on its ability to explain the personal-impersonal distinction. Furthermore, there are a variety of dual-process models that make no particular claims about the role of emotion in deontological moral judgment, either because they do not associate specific processes with specific moral judgments (e.g., as discussed by [35]), or because they dichotomize moral judgments along other lines (e.g., [37–41]). Nonetheless, one highly influential dual-process theory originally argued for by Greene et al. [7] and still frequently discussed by many other researchers, predicts that people’s emotional reactions are causally related to deontological moral judgments and that the signatures of this effect is evident in the personal-impersonal distinction. This is the dual-process theory we aim to test.

### The Present Research

Our goal was to examine the role of emotions in judgments of moral dilemmas using a self-report emotion measure. Self-report measures afford two important benefits: they allow us to identify the specific emotions involved in judgments of moral dilemmas and to assess the strength of the connections between these emotions and moral judgments. There are, of course, limitations to using self-report measures as well. Chief among them is the fact that self-report measures are only suitable when the emotions of interest are available to conscious introspection.

To begin to address this concern, in Experiment 1 we validated our self-report emotion measure by examining people’s emotional responses to the standard battery. Based on the findings of prior neuroimaging studies that have examined people’s judgments about the standard battery, we expected to find significant differences between the emotions elicited by personal and impersonal dilemmas in this battery.

In Experiment 2, we conducted a norming study and confirmed that participants rated personal dilemmas from the standard battery as both more harmful and graphic than impersonal dilemmas. On the basis of these findings, we revised a battery of matched personal and impersonal dilemmas originally created by Moore et al. [26]. We then experimentally confirmed that these dilemmas were matched for harm and graphicness (though they were actually more graphic and harmful on average than the standard battery). Finally, in Experiment 3 we reexamined the role of emotion in moral judgments of dilemmas in the revised battery.

## General Methods

### Participants

Participants were recruited online from Amazon’s Mechanical Turk work distribution website. After recruitment, participants were redirected to a Qualtrics website where the experiment was administered. Before advancing to the experiment, participants indicated consent by clicking a checkbox and could only continue in the experiment if they consented. These experiments were approved by the UCLA Institutional Research Board, IRB 12–000063. Participants were paid $0.60 to participate in Experiments 1 and 3, and $0.75 to participate in Experiment 2. Attention checks were conducted and timings were recorded to ensure participants paid attention while completing the study.

### Materials: Standard Battery

The standard battery, first examined by Greene et al. [7], is composed of 44 moral vignettes describing situations in which a moral decision must be made. Each vignette describes an action which must be taken to avoid an undesirable outcome, but which comes at the cost of another undesirable outcome. These vignettes are divided into two groups: personal moral dilemmas (25 dilemmas) and impersonal moral dilemmas (19 dilemmas). Personal moral dilemmas involve more intimate and direct moral actions than impersonal dilemmas. Within and between these conditions, the vignettes range over a wide variety of situations and actions, from donating to a charitable organization, to pushing a man in front of a train.

### Materials: Revised Battery

We compiled a new set of moral dilemmas to address the concerns we raised about the standard battery (see S1 File). Most of these scenarios were created by modifying the materials originally created by Moore et al. [26]. We modified their materials to further improve the match between personal and impersonal versions of a given dilemma. The revised battery included eight different scenarios (one personal and one impersonal vignette for each scenario for a total of 16 vignettes). In these dilemmas, a moral patient must be harmed in order to maximize utility. For each scenario, a pair of personal and impersonal vignettes were matched as closely as possible, save for the intimacy and directness of the action considered in the vignette. For instance, in the “Space Station” scenario, a fire threatens to break out in the international space station unless a module is vented of oxygen. Unfortunately, an astronaut is trying to exit the module, and his presence in the doorway will prevent the fire safety system from activating. In the personal version of this scenario, the astronaut is stuck in the doorway, and participants must consider whether or not to push him back into the module so that the fire system will be activated. This action will kill him but will save the others on the station. In the impersonal version of the scenario, participants must consider whether to press a switch that will seal the doorway before the astronaut reaches it, with the same consequences as the personal scenario [25].

### Emotion Measure: Positive and Negative Affect Schedule—Expanded Form

In Experiments 1 and 3 we measured participants’ emotional responses to moral dilemmas using scales from the Positive and Negative Affect Schedule expanded form (PANAS-X), a comprehensive emotional state, trait, and mood self-report measure [42, 43]. In our study, we used the PANAS-X as a state emotion measure. The PANAS-X is among the most commonly used self-report measures of emotion (according to Google Scholar, Watson et al.’s paper [43] has been cited over 15,000 times at the time of this writing). The PANAS-X asks participants to rate the extent to which they are experiencing a number of different emotions, on a scale from 1 (very slightly or not at all) to 5 (extremely). The measure is composed of subscales, each of which is composed of several emotion words. We presented participants with the positive and negative affect scales, as well as the guilt, hostility, and joviality scales. Table 1 provides more detail about the variety of emotion words that participants rated.

**Table 1:** Items included in each of the PANAS-X scales.

Negative Affect (10)	afraid, scared, nervous, jittery, irritable, hostile, guilty, ashamed, upset, distressed
Positive Affect (10)	active, alert, attentive, determined, enthusiastic, excited, inspired, interested, proud, strong
Hostility (6)	angry, hostile, irritable, scornful, disgusted, loathing
Guilt (6)	guilty, ashamed, blameworthy, angry at self, disgusted with self, dissatisfied with self
Joviality (8)	happy, joyful, delighted, cheerful, excited, enthusiastic, lively, energetic

*Note*. The number of terms comprising each scale is shown in parentheses.

The Hostility scale includes emotion words like “anger” and “disgust,” which we anticipated would be relevant to moral decisions based on prior research examining norm violations (e.g., [5, 8–12, 28]). We also chose to measure emotional responses using the Guilt scale because the experience of guilt may serve adaptive functions in deterring moral violations and in regulating relationships affected by norm violations (e.g., [44–47]). Finally, the Positive Affect and Joviality scales were used along with the Negative affect scale as general measures of positive and negative affect, respectively.

### Filler Task

We used a filler task to reduce any memory effects caused by repeated administrations of the PANAS-X. Participants were presented with three images taken from the *Where’s Waldo* book series. Participants were asked to search for Waldo, and to click on him when they found him.

### Catch Questions

All experiments included catch questions to identify participants who were not paying attention, or were clicking through the study. These questions were embedded in the response scales, and instructed participants to choose a specific response option. For instance, a catch question embedded in the emotion measure was, “For this item please respond ‘a little’.”

## Experiment 1

As discussed, in Experiment 1 we sought to validate our emotion measure by reproducing prior results demonstrating that personal dilemmas from the standard battery elicit stronger emotional reactions than impersonal dilemmas from the standard battery.

### Participants

We recruited 266 participants to participate in Experiment 1. Of these, 141 participants were female and 125 were male, with mean age of 32.8 years old (*SD* = 11.51).

### Materials

In Experiment 1, moral dilemmas were assigned between-subjects; each participant read one of the 44 moral dilemma vignettes from the standard battery, reproduced verbatim from Greene et al. [7]. Miller & Cushman [48] have argued that action-directed emotions, in particular, are most strongly connected to moral judgments. Consequently, we added an additional sentence to the end of each moral dilemma to direct participants’ attention to the moral action they needed to consider before rating their emotions. For instance, for the “Standard Trolley” dilemma this sentence read, “You are thinking about flipping the switch in order to save the five workmen.”

### Procedure

After collecting demographic information, participants were directed to complete an emotion pre-test. Participants were asked to rate how they were feeling at the present moment using the PANAS-X. This established a baseline for each participant’s emotional state upon entering the study, however this procedure is not necessary to achieve the results we will report henceforth (see [49]). A catch item was included within the emotion scale to ensure participants paid attention. After completing the pre-test, participants completed the filler task (described above).

Participants were then randomly assigned to read a personal or impersonal moral dilemma (participants only read one dilemma). Participants assigned to the personal condition read one of the 25 personal moral dilemmas, and participants in the impersonal condition read one of the 19 impersonal moral dilemmas. Between four and seven participants were assigned to each dilemma (median = 6 per dilemma). After reading a moral dilemma, participants completed the emotion post-test, rating their emotions using the PANAS-X scales a second time. Participants were given specific instructions to rate their emotions as they were currently experiencing them. These instructions read, “Having read the story, how do you feel right now? Please indicate how you actually feel, *not* how you think you might have felt if you were actually in the situation.” Then participants were presented with the PANAS-X scales. Participants generally spent approximately one-minute completing PANAS-X scales each time (pre-test median = 62.5s; post-test median = 64.5s). After rating their emotions, participants were asked to make a moral judgment. They responded using a six-point labeled scale that ranged from “completely inappropriate” to “completely appropriate.” Finally, we asked a final attention check question in which participants were asked whether they took the experiment seriously. The median completion time for the entire experiment was 6 minutes 53 seconds.

### Results and Discussion

Five participants were excluded for missing at least one catch question, leaving 261 participants in the final analyses. The exclusion of these participants did not affect the results of the study.

**Fig 1:**
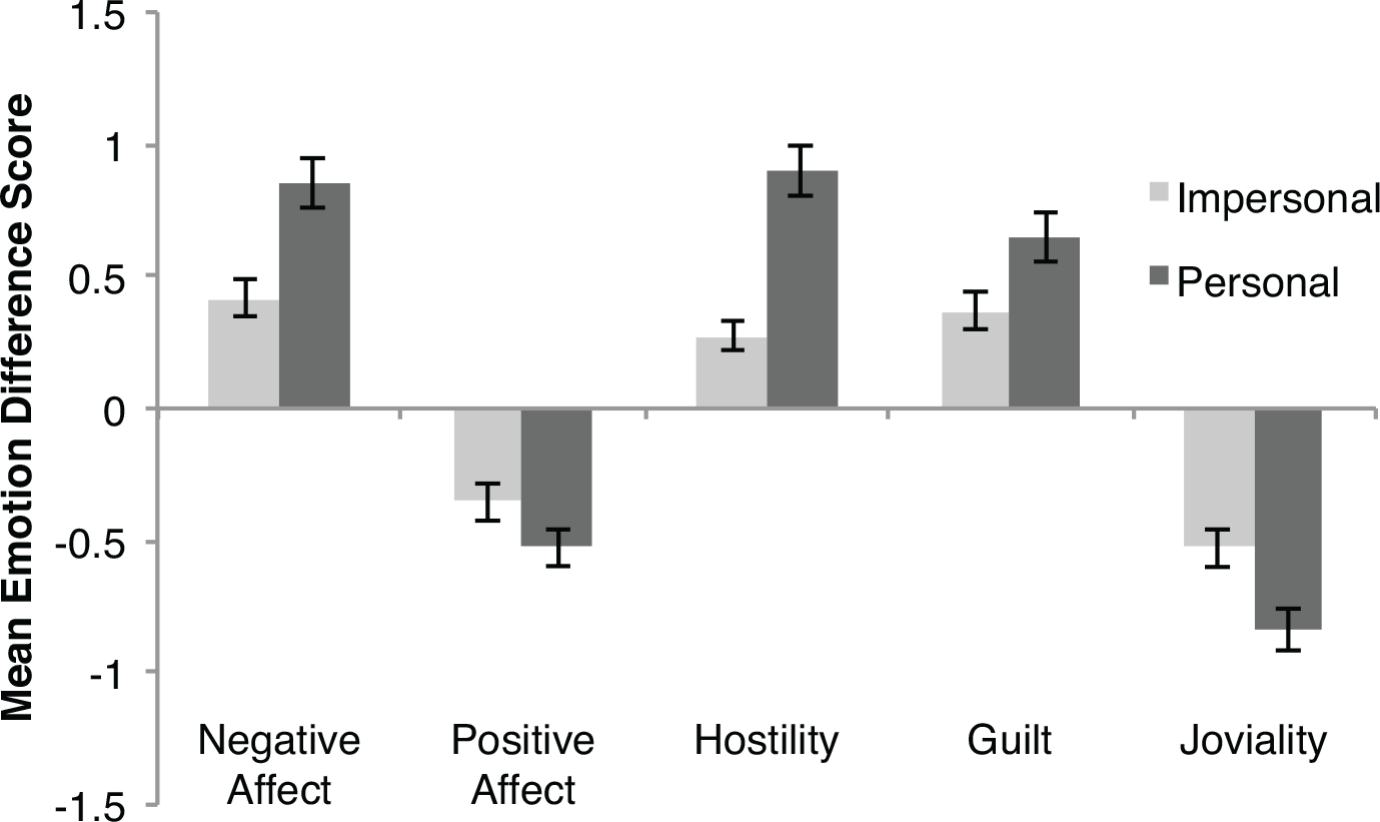
Mean emotion difference scores for PANAS-X subscales across personal and impersonal conditions in Experiment 1. Error bars represent ±1 standard error.

First, we examined participants’ moral judgments, and replicated prior moral psychological findings. Participants made more deontological moral judgments for personal dilemmas (Mean = 2.82, *SD* = 1.915) than for impersonal dilemmas (mean = 3.47, *SD* = 2.00), *t*(259) = -2.67, *p* = .009, 95% *CI* of the difference[.165 to 1.121], *d* = .33). Next, we examined the effect of reading moral dilemmas on participants’ emotional states by computing an *emotional reaction score*. We calculated an emotion reaction score for each subscale by subtracting participants’ pre-test emotion ratings from their post-test emotion ratings on each scale. Mean emotional reaction scores for each condition are shown in Fig 1. Reading both personal and impersonal moral dilemmas led to increased negative emotions (negative affect, guilt, hostility), and decreased positive emotions (positive affect, joviality), when compared to pre-test emotional states.

We conducted a series of 2 x 2 ANOVAs, one for each emotion subscale (summarized in Table 2). We examined two factors with these ANOVAs: a within-subjects Emotional Reaction factor comparing pre-test scores and post-test scores, and a between-subjects Condition factor comparing personal and impersonal dilemmas. Within each ANOVA, the main effect of the Emotional Reaction factor tested whether reading a moral dilemma affected participants’ emotional state, and the interaction between Emotional Reaction and Condition factors tested whether the change in participants’ emotional state was greater for personal dilemmas than for impersonal dilemmas. We observed a significant Emotional Reaction effect for every subscale, indicating that both personal and impersonal dilemmas elicited emotional reactions. We also observed significant interactions between the Emotional Reaction factor and Condition for the negative affect, guilt, hostility, and joviality subscales.

**Table 2:** Summary of ANOVAs conducted on emotion ratings of participants in Experiment 1. The Emotional Reaction factor has been abbreviated as “Reaction.”

		Positive Affect			Negative Affect			Hostility			Guilt			Joviality		
Effect	*df*	*F*	*p*	*η*^*2*^	*F*	*p*	*η*^*2*^	*F*	*p*	*η*^*2*^	*F*	*p*	*η*^*2*^	*F*	*p*	*η*^*2*^
Reaction	1	79.9	< .001	.058	125	< .001	.134	114	< .001	.109	71.5	< .001	.088	148	< .001	.113
Condition	1	8.63	.004	.024	8.67	.004	.019	18.3	< .001	.040	2.66	.104	.006	7.38	.007	.019
Interaction	1	3.04	.082	.002	14.5	< .001	.015	32.6	< .001	.031	5.50	.02	.007	7.73	.006	.006
Error	259															

These interactions indicate that considering a personal dilemma led to significantly greater emotional changes than considering an impersonal dilemma, reproducing the widely reported personal-impersonal emotion effect using a self-report emotion measure. However, we also observed that reading a moral dilemma simpliciter led to increased negative emotions and decreased positive emotions across both personal and impersonal dilemmas, and this effect accounted for the greatest proportion of variance among the effects we examined.

We also tested for correlations between participants’ emotion reaction scores for each emotion scale and their moral judgments. We observed a small but reliable correlation between changes in Hostility and people’s moral judgments (*r* = —.178, *p* = .004). Surprisingly, we observed no reliable correlations between moral judgments and Positive Affect (*r* = .110, *p* = .077), Negative Affect (*r* = —.065, *p* = .294), Guilt (*r* = -.089, *p* = .152), or Joviality (*r* = .063, *p* = .307). If decreased approval for actions in personal dilemmas are a result of stronger emotional reactions, we may expect this relationship to be reflected in correlations between moral judgments and emotional reactions. However, caution should be exercised in interpreting these correlations because, as we have noted, there are other systematic differences between personal and impersonal moral dilemmas in the standard battery that may have attenuated this relationship.

## Experiment 2

Many psychologists and philosophers have argued that people’s stronger emotional responses to personal dilemmas explain the differences in their judgments about personal and impersonal moral dilemmas. However, this interpretation may be unwarranted if emotional differences between these dilemmas can be explained by uncontrolled factors in the standard battery. The most notable difference we observed between personal and impersonal moral dilemmas is the amount of physical harm and graphicness of the descriptions in personal dilemmas. To determine whether this issue was as problematic as we suspected, we performed a norming study on the standard battery and the revised battery.

It should be noted that the findings of this study does not bear on the veracity of the dual-process theory of moral judgment, nor on the role of emotion in the personal-impersonal distinction, nor on the role of emotion in moral judgment more generally. However, identifying confounds in the standard battery may impugn some of the evidence taken to support the dual-process theory of moral judgment.

### Participants

We collected responses from 256 participants for this experiment. Of these, 100 were female and 156 were male. Their mean age was 31.90 years old (*SD* = 10.90).

### Materials and Procedure

After responding to demographic questions, participants were randomly assigned to read four personal and four impersonal vignettes from either the standard battery or from our revised battery of moral dilemmas.

Participants were instructed to first read each vignette and then to rate their agreement with each of the statements that composed the Harm (α = .80) and Graphicness (α = .81) scales (both scales can be found in S1 File). A catch item was included in both scales to ensure participants were paying attention. After participants made these ratings, the vignette remained on the screen while they were asked to make a moral judgment about the vignette. Vignettes were presented in a random order and the ordering of the harm and graphicness scales, as well as the items that compose these scales, were randomized.

For both the harm and graphicness scales, participants were asked to rate their agreement with five statements, two of which were reverse coded. The harm scale included statements such as, “The situation is violent.” The graphicness scale included statements such as, “The language used to describe the situation evokes disturbing images.”

### Results and Discussion

Participants who missed a catch question were excluded from analyses, leaving 215 participants. The decision to exclude these participants did not impact the results of the study. Participants' ratings were averaged to compute harm and graphicness scores for each of the 44 vignettes in the standard battery (mean number of ratings = 29.4) and for the 16 vignettes in the revised battery (mean number of ratings = 26.5).

**Fig 2:**
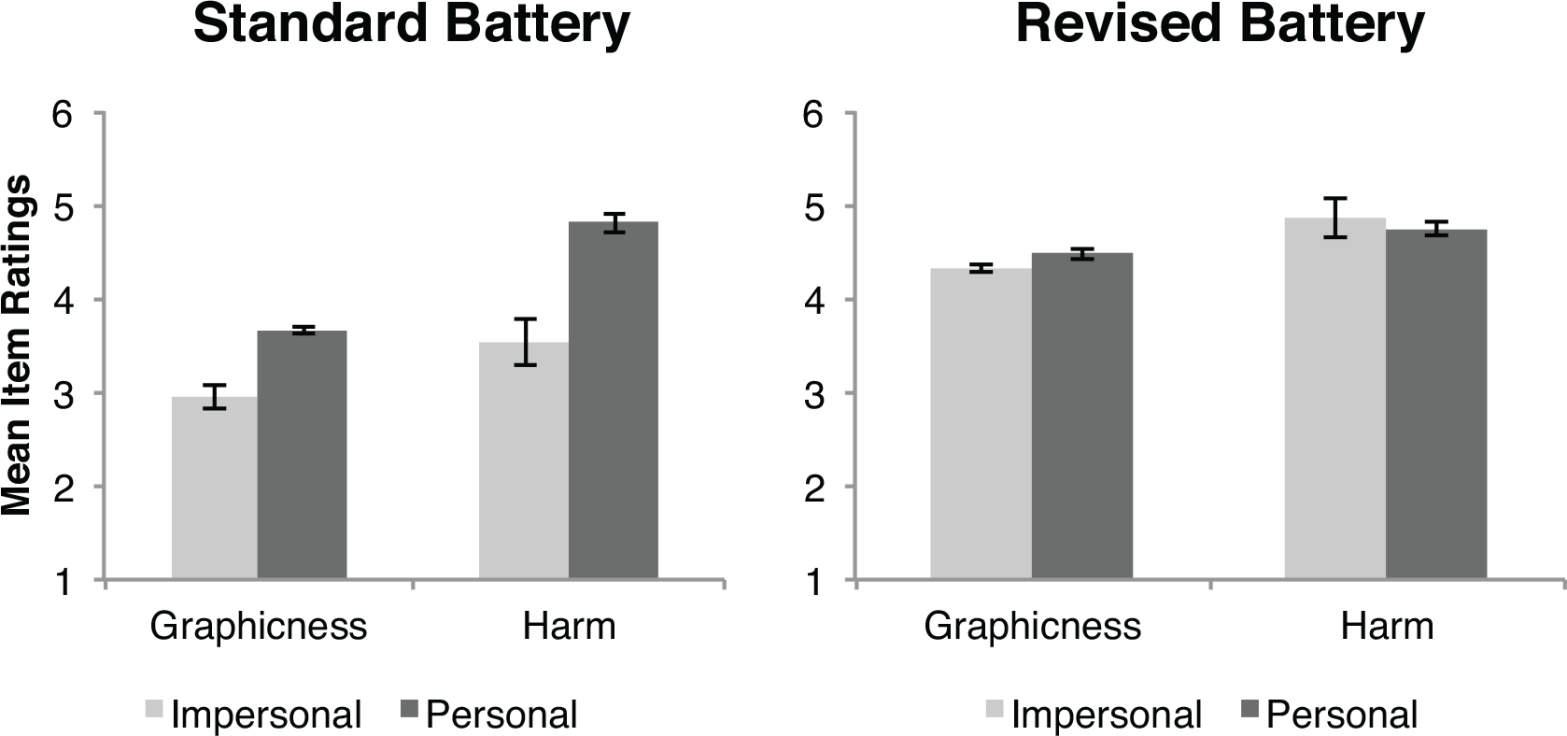
Mean graphicness and harm ratings for items from the standard battery and the revised battery. Error bars represent ±1 standard error.

The results of Experiment 2 are shown in Fig 2. First, we conducted a pair of one-way ANOVAs to determine if the personal dilemmas in the standard battery were viewed as more harmful and more graphic than the impersonal dilemmas. Confirming our predictions, we found that personal dilemmas were viewed as significantly more harmful than impersonal dilemmas, *F*(1,43) = 25.80, *p* < .001, η^2^ = .38. Personal dilemmas were also viewed as more graphic than impersonal dilemmas, *F*(1,43) = 33.66, *p* < .001, η^2^ = .45. In contrast, we observed no significant differences between harm ratings for personal and impersonal dilemmas in the revised battery, *F*(1,15) = .307, *p* = .588, η^2^ = .021. Personal dilemmas tended to be rated as more graphic than impersonal dilemmas, but this trend was not statistically significant, *F*(1,15) = 3.98, *p* = .066, η^2^ = .22.

We also compared the norming ratings for the revised battery with norming ratings of items from the standard battery using a 2 x 2 (condition x battery) ANOVA for each scale (see Fig 2). The harm ratings of both the revised personal and impersonal dilemmas were comparable to the personal dilemmas in the standard battery. We observed significant differences between personal and impersonal moral dilemmas (*F*(1,59) = 6.97, *p* = .011, η^2^ = .111) and between the two batteries (*F*(1,59) = 8.35, *p* = .005, η^2^ = .130). Most importantly, we observed a significant interaction (*F*(1,59) = 10.19, *p* = .002, η^2^ = .154), indicating that the differences between personal and impersonal dilemmas were stronger among the moral dilemmas from the standard battery than among those in the revised battery.

A comparison of graphicness ratings revealed a significant main effect of battery (*F*(1,59) = 93.00, *p* < .001, η^2^ = .624), indicating that our items were *more graphic* than those in the standard battery. An ANOVA also revealed a significant effect of condition (*F*(1,59) = 14.38, *p* < .001, η^2^ = .204), and a significant interaction (*F*(1,59) = 6.48, *p* = .014, η^2^ = .10). The significant interactions in the ANOVAs conducted on harm and graphicness ratings demonstrate that the differences between personal and impersonal dilemmas from the standard battery are not driven by inherent differences in the interpretation of personal and impersonal moral dilemmas, but are more likely due to confounds in the dilemmas of the standard battery.

### Correlational Analyses

As we hypothesized, personal and impersonal dilemmas in the standard battery were not matched for two potentially confounding factors—how harmful the actions were and how graphically the actions were described. In fact, we suspect that these confounding factors are at least partially responsible for participants’ differing emotional reactions to personal and impersonal dilemmas. To test this, we conducted an item-level analysis correlating averaged emotional responses from Experiment 1 with the norming ratings from Experiment 2. Across dilemmas, graphicness scores were significantly correlated with averaged scores for Negative Affect (*r*(42) = .424, *p* = .004, 95% *CI* [.15 to .64]), Hostility (*r*(42) = .450, *p* = .002, 95% *CI* [.18 to .66]), Guilt (*r*(42) = .356, *p* = .018, 95% *CI* [.07 to .59]), and Joviality (*r*(42) = -.391, *p* = .009, 95% *CI* [-.62 to -.11]). Of course, many of the items in the graphicness scale ask about the emotionality of a given moral dilemma, which may partially explain these correlations. However, harm scores also appear to be more weakly correlated with affective responses, although these correlations were not significant across items for Negative Affect (*r*(42) = .277, *p* = .069, 95% *CI* [-.02 to .53]), Hostility (*r*(42) = .213, *p* = .165, 95% *CI* [-.09 to .479]), Guilt (*r*(42) = .233, *p* = .129, 95% *CI* [-.07 to .50]), and Joviality (*r*(42) = -.189, *p* = .218, 95% *CI* [-.46 to .11]).

To overcome the limited power provided by the item analysis, we also examined participant-level correlations among each individual participants’ affective responses in Experiment 1 with the normed ratings for the item that participant was assigned to read. Across participants, harm score ratings were correlated with Negative Affect (*r*(259) = .155, *p* = .011, 95% *CI* [.04 to .27]), Hostility (*r*(259) = .143, *p* = .020, 95% *CI* [.02 to .26]), and Guilt (*r*(259) = .124, *p* = .043, 95% *CI* [.003 to .24]). These correlations were weaker than the item-level correlations, likely because of the additional variability among participants. Finally, we observed significant correlations between an item’s graphicness score and participants’ emotional difference scores on Negative Affect (*r*(259) = .219, 95% *CI* [.10 to .33], *p* < .001), Hostility (*r*(259) = .272, 95% *CI* [.38 to .16], *p* < .001), Guilt (*r*(259) = .170, 95% *CI* [.05 to .29], *p* = .005), and Joviality (*r*(259) = -.165, 95% *CI* [-.28 to -.05], *p* = .007).

Altogether, the results of Experiment 2, which demonstrate that stronger emotional reactions to personal compared to impersonal moral dilemmas are partially explained by differences in the harm and graphicness of personal dilemmas, suggest caution in drawing strong conclusions from prior studies that used the standardized battery.

## Experiment 3

Experiment 2 confirmed that the personal and impersonal moral dilemmas from the revised battery do not differ in their degree of harm or graphicness. In Experiment 3 we examined participants’ reactions to these revised dilemmas, allowing us to test whether personal dilemmas elicit stronger emotional reactions than impersonal dilemmas, as well as the extent to which differences in participants’ emotional responses is predictive of their moral judgments. Recently, some researchers have moved away from explaining differences in people’s responses to personal and impersonal moral dilemmas (e.g., [16, 50]). Instead, their research examines differences in participants’ responses to *high-conflict* personal moral dilemmas. High-conflict dilemmas are thought to be very emotionally evocative and exhibit high levels of disagreement. Given that dilemmas in the revised battery exhibit high levels of disagreement [26] and are also emotionally evocative, in Experiment 3 we are also able to address the role of specific emotions in high-conflict dilemmas.

### Participants

In Experiment 3, we conducted *a priori* power analyses to determine the necessary sample sizes for ANOVA and correlational analyses. We wanted to ensure adequate power to detect effects similar to those observed in Experiment 1. Among the ANOVAs conducted in Experiment 1, the smallest significant effect was observed in participants’ emotion ratings for the Guilt subscale. A power analysis conducted using G*power [51] indicated that a total of 284 participants would be required to achieve 99% power to detect this effect. The Hostility subscale was the only subscale to correlate significantly with participants’ moral judgments in Experiment 1 (*r* = -.178). To detect similar correlations with 99% power, we determined that 568 participants were needed.

In Experiment 3, we recruited 654 participants, anticipating that we might need to remove some participants from our analyses for missing attention check questions. Of these participants, 359 were female and 295 were male. Their mean age was 36.02 years old (*SD* = 13.01).

### Materials and Procedure

In Experiment 3 we used the revised battery, which includes eight moral scenarios, for a total of 16 dilemmas (personal vs. impersonal x 8 scenarios). These dilemmas were assigned between-subjects (for approximately 40 participants per vignette). All other materials and procedures were identical to those used in Experiment 1. Participants spent approximately one-minute completing the PANAS-X scales (pre-test median = 67.0 *s*; post-test median = 71.7 *s*). After completing these procedures, some participants were also asked to answer an additional set of questions about their awareness of their emotional states using the Trait Meta-Mood Measure [52]. We had hoped that this measure might identify those participants for whom emotion and moral judgments would be most strongly connected. However, no differences emerged in these analyses. Therefore, we have omitted discussion of these analyses. The median completion time for Experiment 3 was 10 minutes 46 seconds.

### Results and Discussion

Thirty-eight participants were excluded for missing at least one catch question, or for indicating that they had not paid attention when participating, leaving 616 participants in the final analysis. Our results are unaffected by including these participants in our analyses.

**Fig 3:**
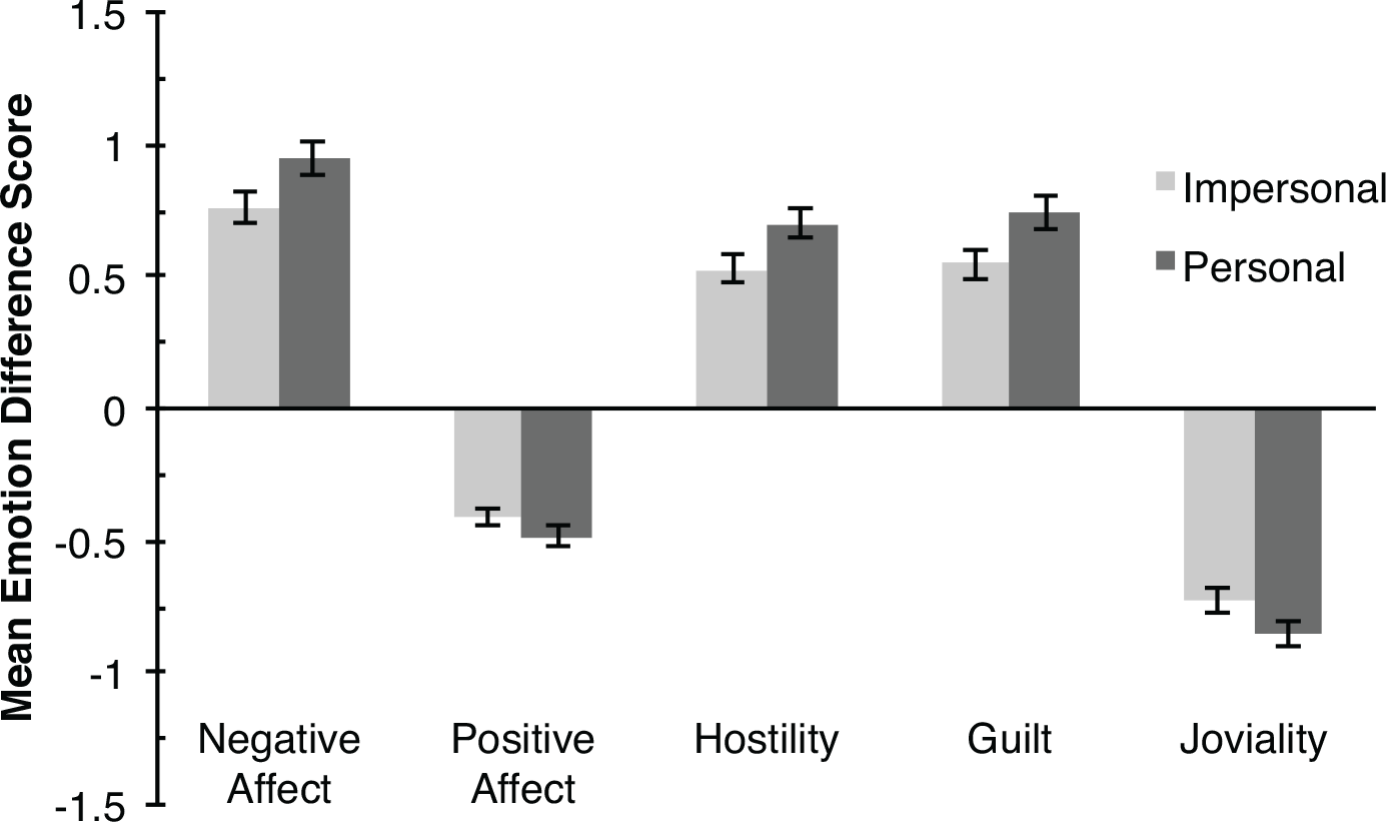
Mean emotion difference scores for PANAS-X subscales across personal and impersonal conditions in Experiment 3. Error bars represent ±1 standard error.

As in Experiment 1, we examined participants’ emotional reactions to the revised battery of moral dilemmas. We calculated an emotional reaction score for participants by subtracting their pre-test emotion ratings from their post-test emotion ratings. Mean differences for participants in each condition are shown in Fig 3. Both personal and impersonal moral dilemmas produced increased negative emotions (Negative Affect, Hostility, and Guilt) and decreased positive emotions (Positive Affect and Joviality).

Just as in Experiment 1, we performed a series of 2 x 2 (Emotional Reaction x Condition) ANOVAs for each emotion subscale (summarized in Table 3). We observed a significant emotional change from pre-test to post-test for every emotion subscale. Then, we tested whether personal dilemmas elicited stronger negative emotional reactions than impersonal dilemmas after matching the dilemmas on graphicness and harm dimensions. To test this, we examined the interaction between the Emotional Reaction and Condition (i.e., personal vs. impersonal) factors. We observed significant interactions between the change in participants’ emotional state and their assigned condition for all subscales except the positive affect and joviality scales, indicating that personal dilemmas still elicited stronger emotional reactions than impersonal dilemmas. These results indicate that personal dilemmas do indeed elicit greater emotional reactions than do impersonal dilemmas, even for the revised battery of moral dilemmas.

**Table 3:** Summary of ANOVAs conducted on emotion rating data from Experiment 3.

		Positive Affect			Negative Affect			Hostility			Guilt			Joviality		
Effect	*df*	*F*	*p*	*η*^*2*^	*F*	*p*	*η*^*2*^	*F*	*p*	*η*^*2*^	*F*	*p*	*η*^*2*^	*F*	*p*	*η*^*2*^
Reaction	1	274	< .001	.064	422	< .001	.198	284	< .001	.113	212	< .001	.124	598	< .001	.177
Condition	1	1.72	.220	.002	2.40	.111	.003	2.12	.146	.002	4.04	.045	.005	1.09	.297	.002
Interaction	1	2.49	.115	.0006	6.89	.009	.004	7.01	.008	.003	6.03	.014	.004	5.47	.020	.002
Error	614															

*Note*. The Emotional Reaction factor has been abbreviated as “Reaction” and interaction terms are labeled using the first letter of each crossed factor.

Although we observed significant differences between the emotions elicited by personal and impersonal dilemmas, these effects were smaller than the effects we observed in Experiment 1. In Experiment 1, the strongest interaction effect we observed was for the Hostility subscale, which accounted for approximately 29% as much variance as the main effect, or a ratio of approximately 3:1. In Experiment 3, the variance accounted for by the Hostility interaction term was less than 3% of that accounted for by the main effect, or a ratio of approximately 35:1. We compared participants’ Hostility reaction scores across Experiments 1 and 3 using a 2 x 2 ANOVA (Condition x Experiment) and found a significant interaction between these experiments (*F*(1, 873) = 10.61, *p* = .001, *η*^*2*^ = .011). This significant effect indicates that differences in the amount of anger and disgust elicited by personal versus impersonal dilemmas were greater in Experiment 1 than 3.

Thus, when examining matched personal and impersonal dilemmas, participant’s emotional states depended much more on whether they had read a moral dilemma than on whether that dilemma was personal or impersonal in nature. How would this affect participant’s moral judgments about these dilemmas? Suggesting a dissociation between participants’ emotional reactions and their moral judgments, we found that participants’ moral judgments were still significantly less approving for personal dilemmas (mean = 2.98, *SD* = 1.750) than impersonal dilemmas (mean = 3.75, *SD* = 1.863), *t*(614) = 5.294, *p* < .001, 95% *CI* [.485 to 1.057], *d* = .42. That is, consistent with prior findings, the personal-impersonal distinction continued to have an important influence on participants’ moral judgments, even though participants’ emotional reactions to these dilemmas were extremely similar. Comparing participants’ moral judgments across Experiments 1 and 3 in a 2 x 2 ANOVA (Condition x Experiment) revealed no significant main effect of Experiment (*F*(1, 873) = 2.467, *p* = .117, η^*2*^ = .002) nor any interaction (*F*(1, 873) = .221, *p* = .639, η^*2*^ = .0002).

Thus, eliminating the confounds in the standard battery did not significantly affect the pattern of moral judgments we observed among personal and impersonal dilemmas. These results suggest that the confounds in the standard battery amplified the differences in emotions elicited by personal and impersonal dilemmas, but had little effect on participants’ moral judgments of these dilemmas.

### Correlational Analyses

We examined the relationship between each participant’s emotional states and their moral judgments. First, we correlated participants’ difference scores for each emotion scale and their moral judgments (see Table 4). We found significant correlations between participants’ moral judgments and the change in their Positive Affect and Hostility scores. The Hostility scale measures emotions like disgust and anger—alarm-bell emotions that researchers have hypothesized are important to moral judgments [e.g., 36]. Thus, our findings lend some support to researchers’ claims about the importance of these emotions relative to emotions like sadness or guilt. However, the size of these correlations are conventionally small, suggesting that the dual-process theory may have placed too great an emphasis on the role of emotion in judgments of personal moral dilemmas. Clearly, more research is necessary to draw firmer conclusions on this matter.

**Table 4:** Correlations between emotion scales and moral judgments after collapsing across conditions in Experiment 3 (above the diagonal). For correlations between moral judgments and the emotion scales, 95% confidence intervals are enclosed in brackets (below the diagonal). Gender is included as a point of reference.

	Negative Affect	Positive Affect	Hostility	Guilt	Joviality	Gender	Moral Judgment
Neg. Affect		-.265**	.823**	.853**	-.473**	.030	-.033
Pos. Affect			-.246**	-.289**	.771**	-.064	.117*
Hostility				.737**	-.399**	.015	-.108*
Guilt					-.441**	-.036	< .001
Joviality						-.066	.023
Gender							-.180*
Moral	[-.11 .05]	[.039 .194]	[-.19 -.03]	[-.08 .08]	[-.06 .10]	[-.25 -.10]	

Note.

*p < .01.

**p < .001.

Coefficients without asterisks *p* > .1

### Mediation analysis

We observed significant changes in participants’ feelings of Hostility between personal and impersonal conditions, and we found that participants’ feelings on this subscale were reliably correlated with their moral judgments. Accordingly, we performed a mediation analysis [53] to test whether differences in moral judgments for personal and impersonal dilemmas can be attributed to increased anger and disgust in response to personal dilemmas. We found a significant indirect effect of condition on moral judgments (10,000 Bootstrapped Samples; Effect: -.0295; 95% *CI*: lower bound = -.0846, upper bound = -.0017). However, the direct effect of condition remained significant (Effect: -.7150; 95% *CI*: lower bound = -1.004, upper bound = -.4258) indicating that emotional reactions only partially mediated the effect of condition on moral judgments. The size of the mediating effect relative to the direct effect is also revealing: although differences in feelings of anger and disgust did account for differences in participants’ moral judgments about personal and impersonal dilemmas (as the correlation suggests), much of this difference is likely still attributable to other factors.

## General Discussion

The present experiments constitute a direct investigation of the role of emotion in people’s judgments of moral dilemmas. Our findings indicate that, as with simple norm violations, anger and disgust play a role in judgments of moral dilemmas. We found that moral dilemmas elicited strong emotional reactions, and that personal dilemmas elicited significantly stronger emotional responses than did impersonal dilemmas. In addition, we found that participants’ experience of anger and disgust were significantly correlated with their moral judgments. However, our findings also suggest that the relationship between emotional reactions and judgments of moral dilemmas is weaker than initially hypothesized. Although the relationship between anger and moral judgment was statistically significant, the correlations between participants’ emotional responses and their moral judgments are conventionally considered small, as was emotion’s mediating effect on participants’ moral judgments.

Clearly, our findings are not the last word on the role of emotion in people’s judgments of moral dilemmas. As discussed, there have been several investigations that seem to demonstrate the role of emotion in moral judgments about simple norm violations (e.g., [5, 8–12]). In addition, recent investigations claim to show a link between emotion and people’s judgments of moral dilemmas using new and more tightly controlled materials that may not suffer from the confounds present in the standard battery (e.g., [50]). Likewise, there is evidence that impairments in empathy can lead to abnormal moral judgments (e.g., [54–56]), suggesting that proper affective functioning is a necessary, but perhaps not a sufficient condition, for making moral judgments. However, our findings still offer important insights on the role of emotion in moral judgment. First, extant research in moral psychology makes it difficult to interpret the strength of the relationship between specific emotional responses and judgments of moral dilemmas. Our findings are novel in this respect, demonstrating a link between emotions like disgust and hostility and moral disapproval in dilemmatic contexts. However, our findings also suggest that this relationship is weaker than anticipated by many researchers. Thus, our findings make room for the possibility that dual-process theories—in particular, those that attempt to explain differences in people’s judgments of personal and impersonal dilemmas by appealing to the emotions these dilemmas elicit—are incomplete.

### Limitations and Future Directions

Self-report emotion measures afford two advantages for assessing the role of emotion in judgments of moral dilemmas. First, these measures allow us to identify the specific emotions experienced during judgments of moral dilemmas. Until now, it was unclear that anger and disgust, rather than guilt, for example, support people’s judgments of moral dilemmas. Second, self-report emotion measures allow us to assess the strength of the relationship between particular emotional responses and people’s moral judgments. These measures also have some clear limitations.

#### Awareness and sensitivity of self-report measures

Self-report measures require participants to be consciously aware of their emotional state and be able to accurately report those states. This raises two potential concerns: First, this limitation prevents our experiments from addressing how unconscious emotional processing may have influenced moral judgments (e.g. [57]). It is clear that further investigation on the link between unconscious emotional responses and judgments of moral dilemmas is warranted, and not at all addressed by the present studies.

Second, even if the relevant emotions are consciously experienced, one might worry whether the PANAS-X are sufficiently sensitive to detect the emotional states that drive moral judgments. Along these lines, one might worry that we used wrong subscales to measure the connection between emotions and moral judgments. Several points speak against the second concern: For one, we not only tested differences between the emotions elicited by personal and impersonal dilemmas, but also tested for differences in participants’ emotions before and after reading a moral dilemma. We observed that reading a moral dilemma had a strong effect on participants’ emotional states. These main effects indicate that the emotions we measured are induced during moral decision-making and that the PANAS-X scales are sufficiently sensitive to detect these effects. Moreover, we provided further validation of our measure by replicating the original emotion differences found in prior research using the items from the standard battery.

#### Disruption of normal operations of emotion and judgment

Another disadvantage to self-report emotion measures is that they explicitly direct participants’ attention toward their emotional responses, whereas in more naturalistic settings participants may experience and be influenced by emotions without explicitly attending to them. This potentially threatens to disrupt the normal connections between emotional responses and moral judgments. If participants are made aware of their emotions they may work to discount them in making their judgments, potentially weakening their connection. Alternatively, participants might feel a demand pressure to make judgments that align with their emotion ratings. Either situation is undesirable from the researcher's perspective. We recognize that ruling out these concerns may require implicit emotion measures that do not direct participants’ attention toward their emotional reactions.

#### Future directions

A number of future directions are suggested by the limitations of prior research, as well as the limitations of the present studies. First, we identified potentially serious confounds in the standard battery of dilemmas that have been used in moral judgment research. As our norming study demonstrates, the revised battery (originally developed by Moore et al. [26]) avoids these confounds and so may be better suited for examining the factors that influence people’s judgments of moral dilemmas.

Future studies might also employ methods capable of measuring unconscious emotional experiences, such as facial expression coding [58] or the measurement of facial muscle activity using electromyography (e.g., [59]). Like galvanic skin response measures (GSR), these methods allow researchers to examine unconscious emotional experiences, yet they also allow researchers to differentiate between different types of emotions. Coupled with carefully controlled and normed materials, these methods might reveal a greater role of unconscious emotions in judgments of moral dilemmas. In addition, these methods would allow researchers to measure emotional reactions without affecting participants’ attention during the decision-making process.

Finally, our findings suggest that occurrent emotions (those experienced during the process of judgment) have only a relatively small role in judgments of moral dilemmas. Still, moral judgments might be more strongly influenced by people’s anticipated emotions, or how people imagine they would feel having taken one or another action (e.g., [60]). Anticipated emotions play an important role in many judgment and decision contexts (e.g., [61–65]), so we might expect that they also influence moral judgments. However, we also think that this idea departs from the claim that alarm bell emotions lead to deontological judgments in personal moral dilemmas (or high-conflict personal dilemmas). Nevertheless, the influence of anticipated emotions in the context of moral dilemmas warrants further examination.

## Conclusion

There has never really been any question as to whether emotions play some role in moral decision-making—even Kant [1, 66] recognized that “sympathies” and “sentiments” are integral to proper moral functioning. Rather, the more substantive concerns are the relative contribution of emotion to people’s moral judgments and whether or not emotion plays an important role in people’s judgments of moral dilemmas. Our findings suggest that emotions (especially anger and disgust) are involved in judgments of moral dilemmas, but that their role in producing these judgments may be weaker than we once thought.

## Supporting Information

S1 File Experimental instructions, materials, and scale items. (DOCX)
