# Supplementary material for: How Large Is the Role of Emotion in Judgments of Moral Dilemmas?
Source: PLoS One. 2016 Jul 6;11(7):e0154780. doi: 10.1371/journal.pone.0154780 (PMC4934695; doi:10.1371/journal.pone.0154780)
Supplement: S1 File — (DOCX) [file pone.0154780.s001.docx]

**Methodology File**

**Experiment 1**

*Instructions*

Please read all instructions and complete this survey in a distraction-free environment. Please avoid taking any breaks while participating in the study.

You will be timed as you progress through the study, and some pages will not allow you to advance until a certain amount of time has passed. This is to ensure that you have sufficient time to read and understand the information presented to you in the study. Your participation in the study is expected to take 15 - 20 minutes.

Please view this survey in a large window with at least 1024 x 768 resolution.

**In this study you will be asked to read a short description of a scenario and make a judgment about that scenario. You will also be asked to rate your emotions.**

*Standard Battery*

**Moral-Impersonal Dilemmas:**

1. Standard Trolley (*1-3*)

You are at the wheel of a runaway trolley quickly approaching a fork in the tracks. On the tracks extending to the left is a group of five railway workmen. On the tracks extending to the right is a single railway workman.

If you do nothing the trolley will proceed to the left, causing the deaths of the five workmen. The only way to avoid the deaths of these workmen is to hit a switch on your dashboard that will cause the trolley to proceed to the right, causing the death of the single workman.

Is it appropriate for you to hit the switch in order to avoid the deaths of the five workmen?

2. Standard Fumes (*2*)

You are the late-night watchman in a hospital. Due to an accident in the building next door, there are deadly fumes rising up through the hospital's ventilation system. In a certain room of the hospital are three patients. In another room there is a single patient. If you do nothing the fumes will rise up into the room containing the three patients and cause their deaths.

The only way to avoid the deaths of these patients is to hit a certain switch, which will cause the fumes to bypass the room containing the three patients. As a result of doing this the fumes will enter the room containing the single patient, causing his death.

Is it appropriate for you to hit the switch in order to avoid the deaths of the three patients?

3. Donation (*3*)

You are at home one day when the mail arrives. You receive a letter from a reputable international aid organization. The letter asks you to make a donation of two hundred dollars to their organization.

The letter explains that a two hundred-dollar donation will allow this organization to provide needed medical attention to some poor people in another part of the world.

Is it appropriate for you to not make a donation to this organization in order to save money?

4. Vaccine Policy (*4*)

You work for the Bureau of Health, a government agency. You are deciding whether or not your agency should encourage the use of a certain recently developed vaccine. The vast majority of people who take the vaccine develop an immunity to a certain deadly disease, but a very small number of people who take the vaccine will actually get the disease that the vaccine is designed to prevent.

All the available evidence, which is very strong, suggests that the chances of getting the disease due to lack of vaccination are much higher than the chances of getting the disease by taking the vaccine.

Is it appropriate for you to direct your agency to encourage the use of this vaccine in order to promote national health?

5. Environmental Policy A1

You are a member of a government legislature. The legislature is deciding between two different policies concerning environmental hazards.

Policy A has a 90% chance of causing no deaths at all and has a 10% chance of causing 1000 deaths. Policy B has a 92% chance of causing no deaths and an 8% chance of causing 10,000 deaths.

Is it appropriate for you to vote for Policy A over Policy B?

6. Environmental Policy A2

You are a member of a government legislature. The legislature is deciding between two different policies concerning environmental hazards.

Policy A has a 90% chance of causing no deaths at all and has a 10% chance of causing 1000 deaths. Policy B has an 88% chance of causing no deaths and a 12% chance of causing 10 deaths.

Is it appropriate for you to vote for Policy B over Policy A?

7. Sculpture (*3*)

You are visiting the sculpture garden of a wealthy art collector. The garden overlooks a valley containing a set of train tracks. A railway workman is working on the tracks, and an empty runaway trolley is heading down the tracks toward the workman.

The only way to save the workman's life is to push one of the art collector's prized sculptures down into the valley so that it will roll onto the tracks and block the trolley's passage. Doing this will destroy the sculpture.

Is it appropriate for you to destroy the sculpture in order to save this workman's life?

8. Speedboat (*3*)

While on vacation on a remote island, you are fishing from a seaside dock. You observe a group of tourists board a small boat and set sail for a nearby island. Soon after their departure you hear over the radio that there is a violent storm brewing, a storm that is sure to intercept them.

The only way that you can ensure their safety is to warn them by borrowing a nearby speedboat. The speedboat belongs to a miserly tycoon who would not take kindly to your borrowing his property.

Is it appropriate for you to borrow the speedboat in order to warn the tourists about the storm?

9. Guarded Speedboat (*3*)

While on vacation on a remote island, you are fishing from a seaside dock. You observe a group of tourists board a small boat and set sail for a nearby island. Soon after their departure you hear over the radio that there is a violent storm brewing, a storm that is sure to intercept them.

The only way that you can ensure their safety is to warn them by borrowing a nearby speedboat. The speedboat belongs to a miserly tycoon who has hired a fiercely loyal guard to make sure that no one uses his boat without permission. To get to the speedboat you will have to lie to the guard.

Is it appropriate for you to lie to the guard in order to borrow the speedboat and warn the tourists about the storm?

10. Five-for-Seven Trolley (*1*-*3*)

You are at the wheel of a runaway trolley quickly approaching a fork in the tracks. On the tracks extending to the left is a group of five railway workmen. On the tracks extending to the right is a group of seven railway workmen.

If you do nothing the trolley will proceed to the left, causing the deaths of the five workmen. The only way to save these workmen is to hit a switch on your dashboard that will cause the trolley to proceed to the right, causing the deaths of the seven workmen on the other side.

Is it appropriate for you to hit the switch in order to avoid the deaths of the five workmen?

11. Three-for-Seven Fumes (*2*)

You are the late-night watchman in a hospital. Due to an accident in the building next door, there are deadly fumes rising up through the hospital's ventilation system. In a certain room of the hospital are three patients. In another room there are seven patients. If you do nothing the fumes will rise up into the room containing the three patients and cause their deaths.

The only way to save these patients is to hit a certain switch, which will cause the fumes to bypass the room containing the three people. As a result of doing this the fumes will enter the room containing the seven patients, causing their deaths.

Is it appropriate for you to hit the switch in order to avoid the deaths of the three patients?

12. Resume

You have a friend who has been trying to find a job lately without much success. He figured that he would be more likely to get hired if he had a more impressive resume.

He decided to put some false information on his resume in order to make it more impressive. By doing this he ultimately managed to get hired, beating out several candidates who were actually more qualified than he.

Was it appropriate for your friend to put false information on his resume in order to help him find employment?

13. Taxes

You are the owner of a small business trying to make ends meet. It occurs to you that you could lower your taxes by pretending that some of your personal expenses are business expenses.

For example, you could pretend that the stereo in your bedroom is being used in the lounge at the office, or that your dinners out with your wife are dinners with clients.

Is it appropriate for you to pretend that certain personal expenses are business expenses in order to lower your taxes?

14. Environmental Policy B1

You are a member of a government legislature. The legislature is deciding between two different policies concerning environmental hazards.

Policy A has a 90% chance of causing no deaths at all and has a 10% chance of causing 1000 deaths. Policy B has a 92% chance of causing no deaths and an 8% chance of causing 10,000 deaths.

Is it appropriate for you to vote for Policy B over Policy A?

15. Environmental Policy B2

You are a member of a government legislature. The legislature is deciding between two different policies concerning environmental hazards.

Policy A has a 90% chance of causing no deaths at all and has a 10% chance of causing 1000 deaths. Policy B has a 88% chance of causing no deaths and a 12% chance of causing 10 deaths.

Is it appropriate for you to vote for Policy A over Policy B?

16. Stock Tip

You are a management consultant working on a case for a large corporate client. You have access to confidential information that would be very useful to investors. You have a friend who plays the stock market. You owe this friend a sizable sum of money.

By providing her with certain confidential information you could help her make a lot of money, considerably more than you owe her. If you did this, she would insist on canceling your debt. Releasing information in this way is strictly forbidden by federal law.

Is it appropriate for you to release this information to your friend so that she will cancel your debt?

17. Illegal Lunch

You are a lawyer working on a big case. The judge presiding over the trial happens to be someone you knew from law school. The two of you were rather friendly back then, but now, decades later, it seems that your old friend barely remembers you.

You're quite sure that if you were to talk to him over lunch, you could jog his memory and he would begin to see you as an old buddy, which would be very good for your work on this case. It's illegal for judges and lawyers working on the same case to meet socially.

Is it appropriate for you to meet with this judge socially in order to help you win your case?

18. Lost Wallet

You are walking down the street when you come across a wallet lying on the ground. You open the wallet and find that it contains several hundred dollars in cash as well the owner's driver's license.

From the credit cards and other items in the wallet it's very clear that the wallet's owner is wealthy. You, on the other hand, have been hit by hard times recently and could really use some extra money. You consider sending the wallet back to the owner without the cash, keeping the cash for yourself.

Is it appropriate for you to keep the money you found in the wallet in order to have more money for yourself?

19. Eyes (*2*)

In the future, you are a veteran of a war during which both of your eyes were destroyed. Due to recent medical advances, it is now possible to perform eye transplants, but, much to your anger and amazement, there are no willing donors.

A sympathetic black-market surgeon offers to help you. If you hire him, he and his associates will kidnap a randomly selected stranger, carve out one of his eyes, and transfer it to you.

Is it appropriate for you to hire this surgeon to carve out a stranger's eye in order to help restore your vision?

**Moral-Personal Dilemmas:**

1. Transplant (*2*)

You are a doctor. You have five patients, each of whom is about to die due to a failing organ of some kind. You have another patient who is healthy.

The only way that you can save the lives of the first five patients is to transplant five of this young man's organs (against his will) into the bodies of the other five patients. If you do this, the young man will die, but the other five patients will live.

Is it appropriate for you to perform this transplant in order to save five of your patients?

2. Footbridge (*2*)

A runaway trolley is heading down the tracks toward five workmen who will be killed if the trolley proceeds on its present course. You are on a footbridge over the tracks, in between the approaching trolley and the five workmen. Next to you on this footbridge is a stranger who happens to be very large.

The only way to save the lives of the five workmen is to push this stranger off the bridge and onto the tracks below where his large body will stop the trolley. The stranger will die if you do this, but the five workmen will be saved.

Is it appropriate for you to push the stranger on to the tracks in order to save the five workmen?

3. Country Road (*3*)

You are driving along a country road when you hear a plea for help coming from some roadside bushes. You pull over and encounter a man whose legs are covered with blood. The man explains that he has had an accident while hiking and asks you to take him to a nearby hospital.

Your initial inclination is to help this man, who will probably lose his leg if he does not get to the hospital soon. However, if you give this man a lift, his blood will ruin the leather upholstery of your car.

Is it appropriate for you to leave this man by the side of the road in order to preserve your leather upholstery?

4. Architect

You are a young architect visiting one of your construction sites with your boss. Your boss is a despicable individual who makes everyone around him miserable including you.

It occurs to you that if you were to push him off of the building you are inspecting he would fall to his death and everyone would think it was an accident.

Is it appropriate for you to push your boss off of the building in order to get him out of your life?

5. Lifeboat*

You are on a cruise ship when there is a fire on board, and the ship has to be abandoned. The lifeboats are carrying many more people than they were designed to carry. The lifeboat you're in is sitting dangerously low in the water-a few inches lower and it will sink.

The seas start to get rough, and the boat begins to fill with water. It seems to you that there is only one way to stop the boat from sinking, and that is to start throwing other passengers overboard, starting with old people who are too weak to resist.

Is it appropriate for you to throw some of your fellow passengers overboard in order to save yourself and the remaining passengers?

6. Hard Times

You are the head of a poor household in a developing country. Your crops have failed for the second year in a row, and it appears that you have no way to feed your family. Your sons, ages eight and ten, are too young to go off to the city where there jobs, but your daughter could fare better.

You know a man from your village who lives in the city and who makes sexually explicit films featuring small children such as your daughter. He tells you that in one year of working in his studio your daughter could earn enough money to keep your family fed for several growing seasons.

Is it appropriate for you to employ your daughter in the child pornography industry in order to feed your family?

7. Smother for dollars

You are in hospital lounge waiting to visit a sick friend. A young man sitting next to you explains that his father is very ill. The doctors believe that he has a week to live at most. He explains further that his father has a substantial life insurance policy that expires at midnight.

If his father dies before midnight, this young man will receive a very large sum of money. He says that the money would mean a great deal to him and that no good will come from his father's living a few more days. He offers you half a million dollars to go up to his father's room and smother his father with a pillow.

Is it appropriate for you to kill this man's father in order to get money for yourself and this young man?

8. Safari*

You and a group of ecologists are studying the wildlife in a remote stretch of jungle when you are captured by a group of paramilitary terrorists. You are held hostage for several days. One of the terrorists takes a liking to you. He informs you that his leader intends to kill you and the rest of the hostages the following morning.

He says that he is willing to help you escape, but that he needs some assurance that you will not go to the police. He devises the following plan. He will videotape you while you kill one of your sleeping colleagues. The tape will ensure your silence, making it possible for you to escape.

Is it appropriate for you to kill your colleague in order to escape from the terrorists and save your own life?

9. Crying Baby

Enemy soldiers have taken over your village. They have orders to kill all remaining civilians. You and some of your townspeople have sought refuge in the cellar of a large house. Outside you hear the voices of soldiers who have come to search the house for valuables.

Your baby begins to cry loudly. You cover his mouth to block the sound. If you remove your hand from his mouth his crying will summon the attention of the soldiers who will kill you, your child, and the others hiding out in the cellar. To save yourself and the others you must smother your child to death.

Is it appropriate for you to smother your child in order to save yourself and the other townspeople?

10. Plane Crash

Your plane has crashed in the Himalayas. The only survivors are yourself, another man, and a young boy. The three of you travel for days, battling extreme cold and wind. Your only chance at survival is to find your way to small a village on the other side of the mountain, several days away.

The boy has a broken leg and cannot move very quickly. His chances of surviving the journey are essentially zero. Without food, you and the other man will probably die as well. The other man suggests that you sacrifice the boy and eat his remains over the next few days.

Is it appropriate to kill this boy so that you and the other man may survive your journey to safety?

11. Hired Rapist

You have been dissatisfied with your marriage for several years. It is your distinct impression that your wife no longer appreciates you. You remember how she appreciated you years ago when you took care of her after she was mugged. You devise the following plan to regain your wife's affection.

You will hire a man to break into your house while you are away. This man will tie up your wife and rape her. You, upon hearing the horrible news, will return swiftly to her side, to take care of her and comfort her, and she will once again appreciate you.

Is it appropriate for you to hire a man to rape your wife so that she will appreciate you as you comfort her?

12. Grandson

A young boy is visiting his grandmother for the weekend. Usually she gives him a gift of a few dollars when he arrives, but this time she doesn't. He asks her why not and she says something about how he doesn't write her as many letters as he used to. Angered by this, the boy decides to play a trick on her.

While his grandmother is busy cooking he removes a handful of pills from the medicine cabinet and puts them in his grandmother's teapot. Later that night, she makes her tea, and the dissolved pills make her sick. In the morning she is dead.

Was it appropriate for this boy to put pills in his grandmother's teapot in order to play a trick on her?

13. Infanticide

You are a fifteen-year-old girl who has become pregnant. By wearing loose clothing and deliberately putting on weight you have managed to keep your pregnancy a secret. One day, while at school, your water breaks. You run to the girls locker room and hide for several hours while you deliver the baby. You are sure that you are not prepared to care for this baby.

You think to yourself that it would be such a relief to simply clean up the mess you've made in the locker room, wrap the baby in some towels, throw the baby in the dumpster behind the school, and act as if nothing had ever happened.

Is it appropriate for you to throw your baby in the dumpster in order to move on with your life?

14. Preventing the Spread*

You are a doctor. One of your patients, whom you diagnosed as HIV positive, is about to be released from the hospital. He has told you, in the confidence of your doctor-patient relationship, that he intends to infect as many people as possible with HIV starting that evening.

Because you are bound by doctor-patient confidentiality, there is no legal way to stop this man from carrying out his plan. It occurs to you that you could contaminate his medication with an untraceable poison that will kill him before he gets a chance to infect others.

Is it appropriate for you to poison this man in order to prevent him from spreading HIV?

15. Modified Lifeboat**

You are on a cruise ship when there is a fire on board, and the ship has to be abandoned. The lifeboats are carrying many more people than they were designed to carry. The lifeboat you're in is sitting dangerously low in the water-a few inches lower and it will sink.

The seas start to get rough, and the boat begins to fill with water. If nothing is done it will sink before the rescue boats arrive and everyone on board will die. However, there is an injured person who will not survive in any case. If you throw that person overboard the boat will stay afloat and the remaining passengers will be saved.

Is it appropriate for you to throw this person overboard in order to save the lives of the remaining passengers?

16. Modified Preventing the Spread**

You are a waiter. You overhear one of your customers say that he is about to go to jail and that in his last forty-eight hours of freedom he plans to infect as many people as possible with HIV. You know him well enough to know that he is telling the truth and that he has access to many potential victims.

You happen to know that he has a very strong allergy to poppy seeds. If he eats even one he will go into convulsions and have to be hospitalized. for at least forty-eight hours.

Is it appropriate for you to cause this man to have a serious allergy attack in order to prevent him from spreading HIV?

17. Modified Safari**

You are part of a group of ecologists who live in a remote stretch of jungle. The entire group, which includes eight children, has been taken hostage by a group of paramilitary terrorists. One of the terrorists takes a liking to you. He informs you that his leader intends to kill you and the rest of the hostages the following morning.

He is willing to help you and the children escape, but as an act of good faith he wants you to kill one of your fellow hostages whom he does not like. If you refuse his offer all the hostages including the children and yourself will die. If you accept his offer then the others will die in the morning but you and the eight children will escape..

Is it appropriate for you to kill one of your fellow hostages in order to escape from the terrorists and save the lives of the eight children?

18. Modified Bomb**

You are negotiating with a powerful and determined terrorist who is about to set off a bomb in a crowded area. Your one advantage is that you have his teen-age son in your custody.

There is only one thing that you can do to stop him from detonating his bomb, which will kill thousands of people if detonated. To stop him, you must contact him over the satellite hook-up that he has established and, in front of the camera, break one of his son's arms and then threaten to break the other one if he does not give himself up.

Is it appropriate for you to break the terrorist's son's arm in order to prevent the terrorist from killing thousands of people with his bomb?

19. Submarine**

You are the captain of a military submarine travelling underneath a large iceberg. An onboard explosion has caused you to lose most of your oxygen supply and has injured one of your crew who is quickly losing blood. The injured crew member is going to die from his wounds no matter what happens.

The remaining oxygen is not sufficient for the entire crew to make it to the surface. The only way to save the other crew members is to shoot dead the injured crew member so that there will be just enough oxygen for the rest of the crew to survive.

Is it appropriate for you to kill the fatally injured crew member in order to save the lives of the remaining crew members?

20. Lawrence of Arabia**

You are the leader of a small army that consists of warriors from two tribes, the hill tribe and the river tribe. You belong to neither tribe. During the night a hill tribesman got into an argument with a river tribesman and murdered him. The river tribe will attack the hill tribe unless the murderer is put to death, but the hill tribe refuses to kill one of its own warriors.

The only way for you to avoid a war between the two tribes that will costs hundreds of lives is to publicly execute the murderer by cutting off is head with your sword.

Is it appropriate for you to cut off this man's head in order to prevent the two tribes from fighting a war that will cost hundreds of lives?

21. Sophie's Choice**

It is wartime and you and your two children, ages eight and five, are living in a territory that has been occupied by the enemy. At the enemy's headquarters is a doctor who performs painful experiments on humans that inevitably lead to death.

He intends to perform experiments on one of your children, but he will allow you to choose which of your children will be experimented upon. You have twenty-four hours to bring one of your children to his laboratory. If you refuse to bring one of your children to his laboratory he will find them both and experiment on both of them.

Is it appropriate for you to bring one of your children to the laboratory in order to avoid having them both die?

22. Sacrifice**

You, your husband, and your four children are crossing a mountain range on your return journey to your homeland. You have inadvertently set up camp on a local clan's sacred burial ground.

The leader of the clan says that according to the local laws, you and your family must be put to death. However, he will let yourself, your husband, and your three other children live if you yourself will kill your oldest son.

Is it appropriate for you to kill your oldest son in order to save your husband and your other three children?

23. Vitamins**

You are the leader of a mountaineering expedition that is stranded in the wilderness. Your expedition includes a family of six that has a genetically caused vitamin deficiency. A few people's kidneys contain large amounts of this vitamin.

There is one such person in your party. The only way to save the lives of the six members of this family is to remove one of this man's kidneys so that the necessary vitamins may be extracted from it. The man will not die if you do this, but his health will be compromised. The man is opposed to this plan, but you have the power to do as you see fit.

Is it appropriate for you to forcibly remove this man's kidney in order to save the lives of the six vitamin-deficient people?

24. Vaccine Test**

A viral epidemic has spread across the globe killing millions of people. You have developed two substances in your home laboratory. You know that one of them is a vaccine, but you don't know which one. You also know that the other one is deadly.

Once you figure out which substance is the vaccine you can use it to save millions of lives. You have with you two people who are under your care, and the only way to identify the vaccine is to inject each of these people with one of the two substances. One person will live, the other will die, and you will be able to start saving lives with your vaccine.

Is it appropriate for you to kill one of these people with a deadly injection in order to identify a vaccine that will save millions of lives?

25. Euthanasia**

You are the leader of a small group of soldiers. You are on your way back from a completed mission deep in enemy territory when one of your men has stepped in trap that has been set by the enemy and is badly injured. The trap is connected to a radio device that by now has alerted the enemy to your presence. They will soon be on their way.

If the enemy finds your injured man they will torture him and kill him. He begs you not to leave him behind, but if you try to take him with you your entire group will be captured. The only way to prevent this injured soldier from being tortured is to shoot him yourself.

Is it appropriate for you to shoot this soldier in order to prevent him from being tortured by the enemy?

*Pre-test Emotion Measure*


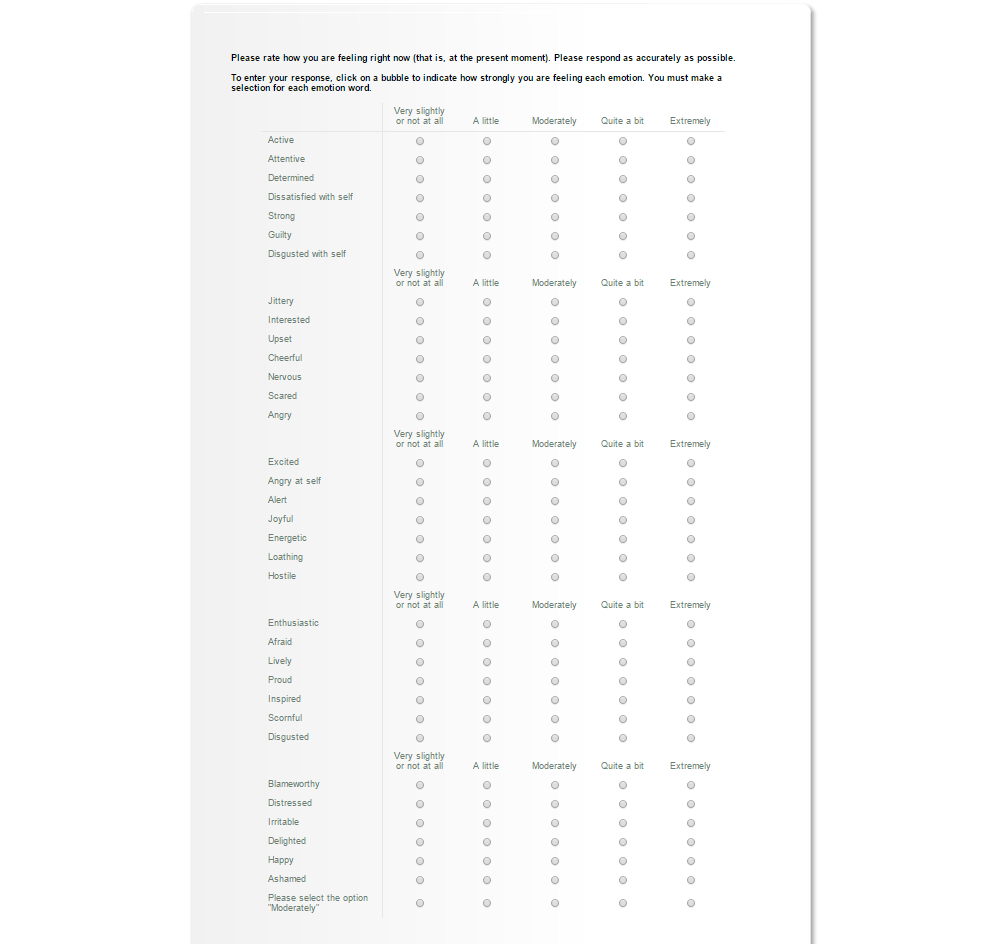


*Post-test emotion measure*


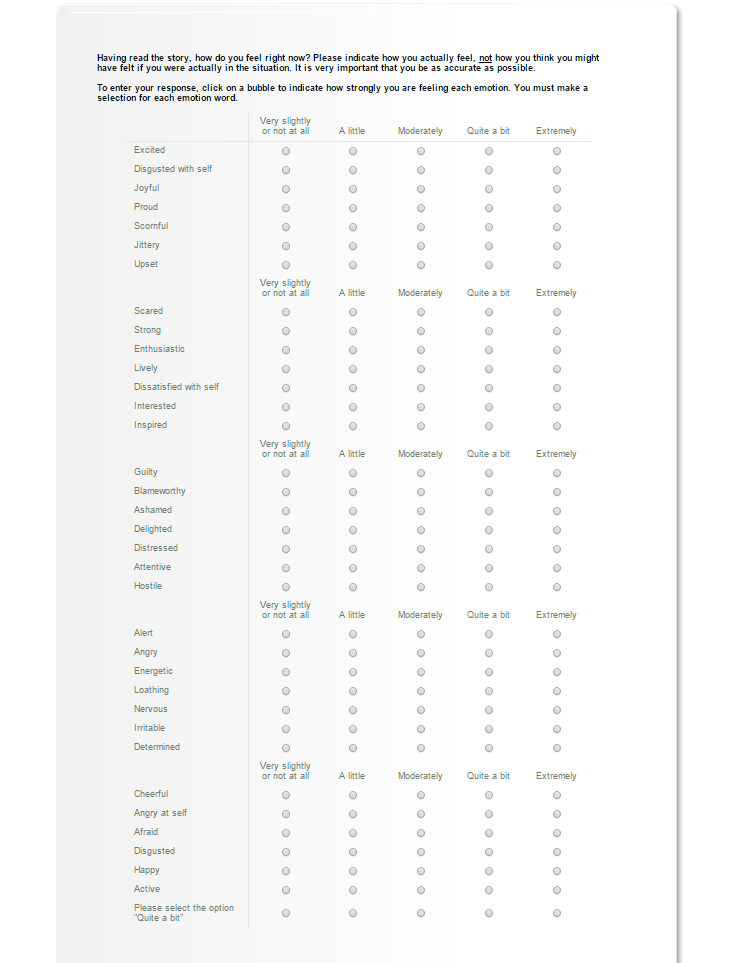


**Experiment 2**

*Instructions*

Please read and follow all instructions carefully. Please complete this survey in a distraction free environment. The survey is expected to take approximately 15 minutes.

In this study you will read short descriptions of 8 different situations where a moral decision must be made. First you will be asked to answer a number of questions about each situation. Then you will make a moral judgment about what should be done in the situation.

Please read each situation carefully and completely before answering questions about it. Some of the situations are similar, but no two are exactly alike, so please read each one. Please answer the questions in the order they appear on screen. Text describing the situation and the questions will appear on the same page, so you may also refer back to the text when answering the questions if you choose.

*Harm Scale*

Participants rated each of these statements on a 6-point Likert scale from Strongly Disagree to Strongly Agree.

1. The situation is violent.
2. In this situation, the threat of injury or death is imminent.
3. This is a peaceful situation.*
4. The action I was considering in this situation would not cause any great harm.*
5. There was a great loss of life in this situation.

*Graphicness Scale*

Participants rated each of these statements on a 6-point Likert scale from Strongly Disagree to Strongly Agree.

1. The description of the situation is highly graphic.
2. The language used to describe the situation evokes disturbing images.
3. The situation is described in emotional and vivid language.
4. The language used to describe the situation seems cold or vague.*
5. It was difficult to picture or imagine the situation.*

** Reverse coded*

**Experiment 3**

Note. Emotion measures were identical to experiment 1.

*Trait Meta-Mood Measure*

TRAIT META-MOOD SCALE

Please read each statement and decide whether or not you agree with it. Place a number in the blank line next to each statement using the following scale.

5 = Strongly agree

4 = Somewhat agree

3 = Neither agree nor disagree

2 = Somewhat disagree

1 = Strongly disagree

__ 1. I try to think good thoughts now matter how badly I feel.

__ 2. People would be better off if they felt less and thought more.

__ 3. I don’t think it’s worth paying attention to your emotions or moods.

__ 4. I don’t usually care much about what I’m feeling.

__ 5. Sometimes I can’t tell what my feelings are.

__ 6. I am rarely confused about what my feelings are.

__ 7. Feelings give direction to life.

__ 8. Although I am sometimes sad, I have a mostly optimistic outlook.

__ 9. When I am upset I realize that the “good things in life” are illusions.

__ 10. I believe in acting from the heart.

__ 11. I can never tell how I feel.

__ 12. The best way for me to handle my feelings is to experience them to the fullest.

__ 13. When I become upset I remind myself of all the pleasures in life.

__ 14. My belief and opinions always seem to change depending on how I feel.

__ 15. I am often aware of my feelings on a matter.

__ 16. I am usually confused about how I feel.

__ 17. One should never be guided by emotions.

__ 18. I never give into my emotions.

__ 19. Although I am sometimes happy, I have a mostly pessimistic outlook.

__ 20. I feel at ease about my emotions.

__ 21. I pay a lot of attention to how I feel.

__ 22. I can’t make sense out of my feelings.

__ 23. I don’t pay much attention to my feelings.

__ 24. I often think about my feelings.

__ 25. I am usually very clear about my feelings.

__ 26. No matter how badly I feel, I try to think about pleasant things.

__ 27. Feelings are a weakness humans have.

__ 28. I usually know my feelings about a matter.

__ 29. It is usually a waste of time to think about your emotions.

__ 30. I almost always know exactly how I am feeling.

*Revised Battery*

**Trolley – Impersonal**

You are at the wheel of a runaway trolley quickly approaching a fork in the tracks. On the tracks extending to the left is a group of five railway workmen. On the tracks extending to the right is a single railway workman. If you do nothing the trolley will proceed to the left, causing the deaths of the five workmen.

The only way to avoid the deaths of these workmen is to hit a switch on your dashboard that will cause the trolley to proceed to the right, causing the death of the single workman.

**Trolley - Personal**

A runaway trolley is heading down the tracks toward five workmen who will be killed if the trolley proceeds on its present course. You are on a footbridge over the tracks, in between the approaching trolley and the five workmen. Next to you on this footbridge is a stranger who happens to be very large.

The only way to save the lives of the five workmen is to push this stranger off the bridge and onto the tracks below where his large body will stop the trolley. The stranger will die if you do this, but the five workmen will be saved.

**Space Station - Impersonal**

You are an engineer on the international space station, in orbit around the Earth. Suddenly, a fire breaks out in the cargo bay. The automatic fire safety system will open the outer door of the cargo bay, letting the oxygen out of the bay and thus putting out the fire. The automatic system only works when the inner portal to the rest of the station has been sealed. One member of the crew, a mechanic in the cargo bay, has put on half of his bulky space suit when the fire begins. He runs to the portal to climb through to safety, but the portal is too narrow to fit through while wearing a space suit.

You realize that if he tries to climb through, he will get stuck and the fire will spread through the rest of the station because the safety system will not operate with the portal open.

There is no time for him to take off the suit before the fire spreads.  

The only way you can prevent the spread of the fire, and save your life and the rest of the crew, is to press a button in the control room that seals the portal before the mechanic gets there.

This way, the system will seal the door and put out the fire, but it will also suck the mechanic into space and kill him.

**Space Station – Personal**

You are an engineer on the international space station, in orbit around the Earth. Suddenly, a fire breaks out in the cargo bay. The automatic fire safety system will open the outer door of the cargo bay, letting the oxygen out of the bay and thus putting out the fire. The automatic system only works when the inner portal to the rest of the station has been sealed. One member of the crew, a mechanic in the cargo bay, has put on half of his bulky space suit when the fire begins. He runs to the portal to climb through to safety, but the portal is too narrow to fit through while wearing a space suit.

You realize that if he tries to climb through, he will get stuck and the fire will spread through the rest of the station because the safety system will not operate with the portal open

There is no time for him to take off the suit before the fire spreads.

The only way you can prevent the spread of the fire, and save your life and the rest of the crew, is to push the mechanic back through the portal and into the cargo bay.

This way, the safety system will seal the door and decompress the cargo bay, but it will also suck the mechanic into space and kill him.

**Transplant - Impersonal**

You are a doctor. You have five patients, each of whom is about to die due to a failing organ of some kind. A new person is rushed into the hospital after a serious car accident. This person is critically injured, and will die without immediate surgery.

While involved in long and complicated surgery on the accident victim, you notice that a nurse is about to give him an inappropriate medication.

You know that this error will be lethal and by ignoring this mistake and allowing it to happen, the patient will die.

This would allow you to harvest his organs for transplant into the bodies of the other five patients.

If you do this, the other five patients will live.

**Transplant – Personal**

You are a doctor. You have five patients, each of whom is about to die due to a failing organ of some kind. A new person is rushed into the hospital after a serious car accident. This person is critically injured, and will die without immediate surgery.

It is likely you can save this accident victim, though it would involve long and complicated surgery.

You realize that if you purposely cut his carotid artery during surgery it would cause his death and no one would ever know.

Then you could harvest his organs for transplant into the bodies of the other five patients.

If you do this, the other five patients will live, but the accident victim will die.

**Rescue Helicopter - Impersonal**

You are the sole paramedic riding on a rescue helicopter, responding to a devastating flood. You have rescued several critically injured people and you have been treating them to keep them alive. Suddenly, the pilot notifies you that there has been an engine malfunction and the helicopter can no longer support the weight of you, the pilot, and all the injured people on board.

If the helicopter crashes, it will kill everyone on board including you, the pilot, and the injured people that you have rescued.

In order to avoid a crash that will kill everyone on the helicopter, you realize that you must lighten the load enough to keep the helicopter aloft.

Because all of the equipment is bolted down, and you are needed by the injured people, the only way to do this is to cut the cable that is raising up the last rescued person.

This will lighten the load enough to keep the helicopter in the air, but it will also kill the person who is dropped.

**Rescue Helicopter - Personal**

You are the sole paramedic riding on a rescue helicopter, responding to a devastating flood. You have rescued several critically injured people and you have been treating them to keep them alive. Suddenly, the pilot notifies you that there has been an engine malfunction and the helicopter can no longer support the weight of you, the pilot, and all the injured people on board.

If the helicopter crashes, it will kill everyone on board including you, the pilot, and the injured people that you have rescued.

In order to avoid a crash that will kill everyone on the helicopter, you realize that you must lighten the load enough to keep the helicopter aloft.

Because all of the equipment is bolted down, and you are needed by the injured people, the only way to do this is to throw one of the injured people off the helicopter.

This will lighten the load enough to keep the helicopter in the air but will cause the death of the person thrown off.

**Deep Sea Lab – Impersonal**

You are an engineer in the International Deep Sea Research Lab. Suddenly, a fire breaks out in the cargo bay. The automatic fire safety system will open the outer door of the storage bay, letting water into the bay and thus putting out the fire. The automatic system only works when the inner portal to the rest of the research lab has been sealed. One member of the crew, a mechanic in the storage bay, has put on half of his bulky deep sea suit when the fire begins. He runs to the portal to climb through to safety, but the portal is too narrow to fit through while wearing a deep sea suit.

You realize that if he tries to climb through, he will get stuck and the fire will spread through the rest of the lab because the safety system will not operate with the portal open.

There is no time for him to take off the suit before the fire spreads.

The only way you can prevent the spread of the fire, and save your life and the rest of the crew, is to press a button in the control room that seals the portal before the mechanic gets there.

This way, the system will seal the door and put out the fire, but the pressure of the water will crush the mechanic and kill him.

**Deep Sea Lab – Personal**

You are an engineer in the International Deep Sea Research Lab. Suddenly, a fire breaks out in the storage bay. The automatic fire safety system will open the outer door of the storage bay, letting water into the bay and thus putting out the fire. The automatic system only works when the inner portal to the rest of the research lab has been sealed. One member of the crew, a mechanic in the storage bay, has put on half of his bulky deep sea suit when the fire begins. He runs to the portal to climb through to safety, but the portal is too narrow to fit through while wearing a deep sea suit.

You realize that if he tries to climb through, he will get stuck and the fire will spread through the rest of the lab because the safety system will not operate with the portal open.

There is no time for him to take off the suit before the fire spreads.

The only way you can prevent the spread of the fire, and save your life and the rest of the crew, is to push the mechanic back through the portal and into the storage bay.

This way, the safety system will seal the door and allow water into the storage bay, but the pressure of the water will crush the mechanic and kill him.

**Ebola - Impersonal**

You are a Peace Corps health-worker who is volunteering in a rural African village. A prominent man from a nearby village has contracted an Ebola virus that is extremely contagious, incurable, and almost always fatal within a week. 

Miraculously, this man has survived for a month, and so he must be a rare carrier who is immune to the virus’ deadly effects. However, this man wrongly believes that your health center can cure his disease.
 
You see him approaching and you know that if he enters the village he will spread the virus to hundreds of innocent people who, unlike him, will die.
 
The only way you can prevent him from entering the village and spreading the virus to you and the rest of the village is to tell the village sniper to shoot and kill him as he approaches.

**Ebola - Personal**

You are a Peace Corps health-worker who is volunteering in a rural African village. A prominent man from a nearby village has contracted an Ebola virus that is extremely contagious, incurable, and almost always fatal within a week. 

Miraculously, this man has survived for a month, and so he must be a rare carrier who is immune to the virus’ deadly effects. However, this man wrongly believes that your health center can cure his disease.
 
You see him approaching and you know that if he enters the village he will spread the virus to hundreds of innocent people who, unlike him, will die.
 
There is a loaded gun in the health center.
 
You realize that the only way you can prevent him from entering the village and spreading the virus to you and the rest of the village is to shoot and kill him as he approaches.

**Fumes – Impersonal**

You are an orderly in a hospital during the night shift. You notice that a chemical has just been spilled in a room containing five patients. This chemical is highly toxic and if left on the floor will rapidly evaporate creating a poisonous gas.
 
This will inevitably kill all the patients in the room and will also kill a single patient next door, as the two rooms share a ventilation system. This chemical cannot simply be mopped up, nor can it be soaked up with towels or sheets.
 
However, you can flip a switch controlling the ventilation system that will draw the poisonous fumes out of the room in which the spill has occurred, and into the room next door, saving the five patients.
 
Unfortunately, this will quickly kill the patient in the neighboring room before anyone can help her.

**Fumes – Personal**

You are an orderly in a hospital during the night shift. You notice that a chemical has just been spilled in a room containing six patients. This chemical is highly toxic and if left on the floor will rapidly evaporate creating a poisonous gas. This will inevitably kill all the patients in the room.
 
This chemical cannot simply be mopped up, nor can it be soaked up with towels or sheets. However, because you have worked around this chemical before, you know that this chemical absorbs into human skin very rapidly.
 
You could pull one of the patients out of bed onto the spill so that the chemical will completely soak into her skin instead of evaporating into the air in the room.
 
This will quickly kill the one patient but save the other five patients from the poisonous gas.

**Rescue Boat – Impersonal**

You are the sole paramedic riding on a rescue boat, responding to a devastating flood. You have rescued several critically injured people and you have been treating them to keep them alive. Suddenly, the captain notifies you that the boat's hull has been damaged by debris and the boat can no longer support the weight of you, the captain, and all the people you are trying to rescue without sinking.

If the boat sinks, everyone on board including you, the captain, and the injured people that you have rescued will drown in the strong currents of the flood.

In order to avoid the boat sinking, you realize that you must lighten the load enough to keep the boat from sinking.

Because you are needed by the injured people, the only way to do this is to cut the cable that is towing the last injured person in.

Preventing him from getting in the boat will keep the boat from sinking, but it will also kill the person who you stopped towing in.

**Rescue Boat – Personal**

You are the sole paramedic riding on a rescue boat, responding to a devastating flood. You have rescued several critically injured people and you have been treating them to keep them alive. Suddenly, the captain notifies you that the boat's hull has been damaged by debris and the boat can no longer support the weight of you, the captain, and all the people you are trying to rescue without sinking.

If the boat sinks, everyone on board including you, the captain, and the injured people that you have rescued will drown in the strong currents of the flood.

In order to avoid the boat sinking, you realize that you must lighten the load enough to keep the boat from sinking.

Because you are needed by the other injured people, the only way to do this is to push one of the injured people off the boat.

Pushing him off the boat will keep the boat from sinking, but it will also kill the person who you pushed overboard.
